# Monitoring SARS‐CoV‐2 Dissemination in Wastewater and Virus Isolation in Cell Cultures: An Integrated Approach for Pathogen Detection and Surveillance

**DOI:** 10.1111/jcmm.70805

**Published:** 2025-09-01

**Authors:** Elena Radu, Tudor Emanuel Fertig, Laura Denisa Dragu, Ioana Mădălina Pitică, Marius Surleac, Ana Iulia Neagu, Lavinia Pană, Alexandra Păiş, Lilia Matei, Ionut‐Lucian Antone‐Iordache, Daciana Silvia Marta, Victor‐Eduard Peteu, Mihai Niţă‐Lazăr, Cătălina Stoica, Cornel Popescu, Camelia Mădălina Sultana, Anca Botezatu, Iulia Virginia Iancu, Mihaela Chivu‐Economescu, Leontina Banică, Ana Sorinica Petre, Simona Paraschiv, Mihaela Gherghiceanu, Simona Maria Ruta, Norbert Kreuzinger, Carmen Cristina Diaconu, Coralia Bleotu

**Affiliations:** ^1^ Department of Cellular and Molecular Pathology Stefan S. Nicolau Institute of Virology Bucharest Romania; ^2^ Institute for Water Quality and Resource Management Vienna University of Technology Vienna Austria; ^3^ Carol Davila University of Medicine and Pharmacy Bucharest Romania; ^4^ Ultrastructural Pathology and Bioimaging Lab Victor Babeș National Institute of Pathology Bucharest Romania; ^5^ Research Institute of the University of Bucharest‐ICUB University of Bucharest Bucharest Romania; ^6^ National Institute of Infectious Diseases Prof. Dr. Matei Balș Bucharest Romania; ^7^ National Research and Development Institute for Industrial Ecology (ECOIND) Bucharest Romania; ^8^ Victor Babes Clinical Hospital of Infectious and Tropical Diseases Bucharest Romania; ^9^ Apa Nova WasteWater Treatment Plant Directorate Bucharest Romania; ^10^ Academy of Romanian Scientists Bucharest Romania

**Keywords:** environmental surveillance, SARS‐CoV‐2 infectivity, virus isolation, wastewater monitoring

## Abstract

Our study presents wastewater (WW) monitoring data, focusing on determining the infectivity of SARS‐CoV‐2 in the collected samples. Additionally, a panel of different viruses has been tested in the WW samples. The untreated WW monitoring campaign took place over 1 year in Bucharest, with approximately 300 samples being collected twice a week at the wastewater treatment plant (WWTP) and an infectious diseases hospital. Our data indicated that SARS‐CoV‐2 concentrations in WW preceded the increase in the number of clinical cases by nearly 2 weeks. Differences between locations were notable, with higher raw concentrations in WW samples from the hospital than those from the WWTP. However, after normalising to population equivalent, the hospital samples concentrations dropped significantly, suggesting that WW monitoring at the urban level provides a more complete and epidemiologically relevant picture than data obtained only from hospitals. Only a few isolates could demonstrate SARS‐CoV‐2 persistence during in vitro passages. Although the success rate was low, the technique remains crucial for validating the viability of viruses. Adenovirus, Bocavirus and Reovirus were the most abundant ones in both urban and hospital wastewater. WW monitoring remains the most effective approach for tracking the dissemination of various pathogens and supporting public health authorities.

## 1 Introduction

Urban untreated wastewater (WW) contains a considerable diversity and high abundance of pathogenic viruses [1, 2, 3, 4], which reflects infection patterns in the human population responsible for waterborne diseases and the associated symptoms, including gastrointestinal conditions and respiratory diseases [3]. Consequently, it is essential to monitor viruses in WW for several purposes, including assessing the risk associated with their spread through water, evaluating the effectiveness of disinfection as a control measure and tracking their dissemination in the population [5]. To achieve these goals, it is crucial to determine the concentration and infectivity of viruses. Since viruses require a host species for replication, their absence may lead to a reduction in their concentration in the environment through progressive inactivation [3]. Coronaviruses have been reported in WW from various sources; the main sources of infection are represented by viral RNA shedding in faeces [6], as well as through handwashing, sputum or vomiting [7]. Moreover, the presence of highly pathogenic coronaviruses in urine was also reported, i.e., SARS‐CoV [8], MERS [9] and lately SARS‐CoV‐2 [10]. Wastewater‐based epidemiology (WBE) has been shown to be a powerful tool for monitoring the dissemination of SARS‐CoV‐2 and its circulating variants in a defined catchment [1, 11, 12, 13, 14, 15]. There is explicit evidence that SARS‐CoV‐2 was first identified in WW inflow samples before clinical reporting data [1, 11, 12, 15].

SARS‐CoV‐2 detection in faeces and wastewater was largely reported using RT‐PCR, considered the gold standard method worldwide, but little is known about its ability to replicate and transmit the infection. Therefore, other approaches are also needed to detect the virus's viability and replicative capacity. The most important and widely used approaches are represented by virus isolation in cell cultures [16], detection of viral particles by transmission electron microscopy (TEM) [8, 17] and whole genome sequencing for the identification of the circulating strain [18]. Culturing SARS‐CoV‐2 remains challenging, given the limited knowledge on its persistence in WW and the infectious potential of such samples [19]. Using correlative light microscopy and TEM [20] and observing the presence of intact and degraded SARS‐like particles, the viral infectivity was inconclusive. Few studies have detected intact SARS‐CoV‐2 virions in faeces capable of completing replication cycles. Reports indicate no evidence of viability and infectivity of SARS‐CoV‐2 in the faecal specimens of COVID‐19 patients [21].

The main objectives of the present manuscript are represented by longitudinal WW monitoring campaign in Bucharest, Romania and the isolation of viable SARS‐CoV‐2 virions from untreated WW samples using cell culture employing various virological diagnostic methods to demonstrate the viability of the virus in this type of biological sample and suggest its potential infectivity. Using WW data and its correlation with epidemiological reports, as well as different SARS‐CoV‐2 sub‐lineages isolated in cell culture, the manuscript focuses on supporting public health authorities.

## 2 Materials and Methods

### 2.1 Collection and Concentration of Viral Particles in Untreated WW Samples

The longitudinal monitoring WW campaign took place in Bucharest, Romania and samples were collected from the main WWTP (24‐h flow‐weighted composite and grab samples) and a hospital of infectious diseases (grab sample) between October 18, 2022 and November 28, 2023, twice a week for 55 weeks. The samples were kept at 4°C for the whole sampling period and delivered to the laboratory within 2–4 h after collection. Total SARS‐CoV‐2 viral particle concentrations were carried out using the PEG precipitation protocol previously described by Radu et al. [11]. Briefly, 45 mL wastewater aliquots were centrifuged for debris removal, and viral particles were precipitated with 100 g/L PEG 8000 (Sigma Aldrich) and 22.5 g/L NaCl (Sigma Aldrich) and centrifugation. The pellet was resuspended in 400 μL molecular biology grade water (Sigma Aldrich), 400 μL CTAB (Promega) buffer and 40 μL of Proteinase K (Promega) and run on a FastPrep24 machine for 40 s at 6 m/s. The RNA extraction was performed on Maxwell RSC AS4500 (Promega) device using the Viral RNA/DNA Extraction Kit from WW (Promega). Total viral RNA was eluted in 100 μL TE buffer and stored at −80°C for downstream analyses. Each WW sample was spiked with a known concentration of the internal positive control IPC (6 × 10^5^ copies/μL) prior to RNA extraction, and the recovery efficiency was calculated as follows: Recovery efficiency [%] = (IPC recovered/IPC seeded) × 100. Furthermore, the RT‐qPCR inhibition was determined by comparing the IPC Cq (quantification cycle) of the respective spiked WW sample with the IPC Cq value of a non‐template control sample (containing molecular biology water instead of RNA extract). A threshold difference of ≤ 2 cycles indicated no inhibition [22]. The recovery average rate was determined as 37.72%. A threshold Cq value of 35 cycles was implemented to consider a positive sample and use it further for WGS (Whole Genome Sequencing). Two representative samples from each month and each location were subjected to sequencing to determine the circulating variants at that specific time point in the selected location.

### 2.2 Assessment and Quantification of SARS‐CoV‐2 Viral Particles Through RT‐qPCR in WW Samples

WW SARS‐CoV‐2 concentration was determined by targeting the highly conserved region of the nucleocapsid protein gene (N gene) on a Biorad CFX96 instrument (BioRad). One‐step RT‐qPCR analysis was performed using the ViroReal Kit SARS‐CoV‐2 & SARS (Ingenetix, Vienna, Austria) in a total volume of 20 μL/reaction, according to the manufacturer's instructions. The average PCR efficiency was calculated as 99.82% and all tested samples were considered valid (Cq value < 35 cycles).

### 2.3 SARS‐CoV‐2 Digital PCR

The accurate detection and quantification of viral genetic material in complex WW samples were achieved using the Absolute Q dPCR SARS‐CoV‐2 Wastewater Surveillance Kit (Thermo Fisher Scientific, USA) on 72 wastewater samples, according to the manufacturer's instructions. The kit contains primers and probes to detect two different regions of the SARS‐CoV‐2 N gene (N gene region 1 and N gene region 2) and primers for Pepper Mild Mottle Virus (PMMoV) to normalise the results of N gene targets to the amount of human faecal material in the WW sample. Results were reported as genome copies of viral RNA per microlitre of WW (gc/μL).

### 2.4 Detection of Other Respiratory Viruses

In total 72 extracts from the untreated WW samples were tested for other respiratory viruses using the Respiratory Pathogens Panel Kit v7 (Anatolia Geneworks, Bosphore, Turkey). Real‐time PCR amplification was performed according to the manufacturer's specifications using the CFX96 Touch Real‐Time PCR Detection System (Bio‐Rad, USA). Fluorescence from specific probes enabled pathogen detection, with Ct < 28 for positive controls and Ct ≤ 32 for the internal control as acceptance criteria.

### 2.5 Reoviruses Detection

Minor adjustments of the nested PCR method described by Wellehan Jr. et al. were used to detect Reoviruses [23]. High‐Capacity cDNA Reverse Transcription Kit (Thermo Fisher Scientific, USA) was used for reverse transcription of 10 μL WW RNA, and Maxima SYBR Green/ROX qPCR Master Mix (Thermo Fisher Scientific, USA) for nested PCR. The thermocycler amplification programme for the first PCR was as follows: initial denaturation at 95°C for 10 min, 45 cycles of denaturation at 95°C for 1 min, primer annealing at 52°C for 1 min and final DNA extension at 72°C for 1 min. The second round of PCR had the following programme: initial denaturation at 95°C for 10 min, followed by 45 cycles of denaturation at 95°C for 1 min, primer annealing at 47°C for 1 min, DNA extension at 72°C for 1 min, followed by a final extension step at 72°C for 8 min. For confirmation, PCR products were visualised in 1% agarose gel.

### 2.6 SARS‐CoV‐2 WGS in Untreated WW Samples

To identify the circulating variants of SARS‐CoV‐2 in the collected WW samples, a sequencing protocol based on the WGS technique was used. The sequencing of 73 untreated WW samples was performed on the MiSeq equipment from Illumina using the COVIDSeq Assay Index kit and MiSeq Reagent Kit V3 (600‐cycle). Briefly, library preparation consisted of one step of reverse transcription of SARS‐CoV‐2 RNA into cDNA, followed by specific amplification of the viral genome using cocktails of primers (ARTIC v4) [24]. The generated 99 amplicons were further treated as recommended by Illumina technology (tagmentation, barcoding, pooling and denaturation prior to loading to the sequencer). The SARS‐CoV‐2 raw fastq.gz files underwent quality control (QC) trimming, reference mapping assembly and lineage prediction using the HAVoC pipeline [25]. Additionally, lineage assignment was conducted using the Pangolin web page (https://pangolin.cog‐uk.io) [26]. Among the consensus sequences generated, 14 sequences could not be assembled due to insufficient coverage across the entire SARS‐CoV‐2 genome length. The consensus sequences were compared against the GISAID database (https://gisaid.org) [27] via BLAST for phylogenetic analysis, with closely matching hits (> 99.8% identity) further retrieved. All SARS‐CoV‐2 sequences were aligned using MAFFT [28]. Subsequently, a bootstrapped phylogenetic tree was constructed using RA×ML [29] and visualised using FigTree (http://tree.bio.ed.ac.uk/software/figtree/) [30]. The phylogenetic tree image was then exported using Affinity Designer.

### 2.7 SARS‐CoV‐2 Isolation on Cell Cultures

The pellet obtained after the PEG precipitation method was resuspended in 2 mL SM buffer (200 mM NaCl_2_, 10 mM MgSO_4_, 50 mM Tris–HCl, pH 7.5) for virus isolation on cell culture. VeroE6‐C1008 (ATCC No. CRL‐1586) cell lines were used for virus isolation. The cells were seeded in specialised culture tubes for virus cultivation at a concentration of 2.5 × 10^5^ cells/tube in Dulbecco's Modified Eagle Medium (DMEM, Thermo Fisher Scientific) supplemented with 10% foetal bovine serum (FBS, Euroclone). One mL of WW viral suspension, previously filtered through a 0.22 μm pore‐size filter, was added over the cell monolayer and incubated at 37°C for 60 min. After adsorption, a media with 2% FBS was added. The culture tubes were incubated at 37°C in a 5% CO_2_ atmosphere and were monitored for a minimum of 5 days post‐infection. Cell morphology was compared to that of a control, uninoculated culture. When the cytopathic effect (CPE) was observed, three rounds of freeze–thaw were performed and the viral supernatant was aliquoted for long‐term storage at −80°C. Viral isolation experiments were performed on all collected samples from both locations in the Biosafety Level 3 laboratory (BLS3) under the CDC's good practices [31].

### 2.8 Transmission Electron Microscopy

For transmission electron microscopy (TEM), 5 × 10^5^ VeroE6 cells were plated in a 25 cm^2^ culture flask and inoculated with SARS‐CoV‐2 isolates from WW. When approximately two‐thirds of the cell monolayer showed signs of infection, media was changed with a pre‐warmed fixative comprising 0.1 M cacodylate buffer (pH 7.4) with 2.5% glutaraldehyde and 1.4% sucrose. After overnight incubation at 4°C, cells were scraped and prepared as described elsewhere [32]. Briefly, cells were post‐fixed with buffered 1% OsO_4_, then embedded in epoxy resin (Agar100 Resin Kit, Agar Scientific, Essex, UK). Ultra‐thin sections (40–60 nm) were mounted on carbon and Formvar‐coated copper grids, then stained with 1% uranyl acetate and Reynolds lead citrate (Agar Scientific, Essex, UK). Micrographs were recorded by the 4 K × 4 K Ceta camera of a 200 kV Talos F200C TEM (Thermo Fisher Scientific, Waltham, MA, USA). For each sample, an average of 100 cell sections was evaluated for signs of infection.

## 3 Results

### 3.1 Monitoring of SARS‐CoV‐2 Viral Particles in Untreated WW Samples

SARS‐CoV‐2 RNA concentration in untreated WW was higher in the grab sample collected from the Hospital (6.5 log gc/L) compared to the grab and composite samples collected from the WWTP (5.5–5.7 log gc/L). The untreated WWTP grab sample showed concentrations of SARS‐CoV‐2 viral particles similar to the composite sample, suggesting that when it is not possible to collect (for technical, organisational reasons, resources, equipment, etc.) a composite sample, it can be replaced with the grab one, with the remark that it should be collected at peak hours to be representative.

SARS‐CoV‐2 concentration quantified using dPCR displays a similar profile, the concentration of SARS‐CoV‐2 RNA using the first region of the N gene as target was 3.49E+08 gc/μL in the untreated grab WW/from the hospital, respectively 2.10E+07 gc/μL and 1.34E+07 gc/μL in untreated composite and grab WWTP, and for the second region of the N gene 2.28E+08 gc/μL in grab untreated WW from the hospital respectively 1.79E+07 and 1.13E+07 gc/μL in untreated composite and grab WWTP.

**FIGURE 1:**
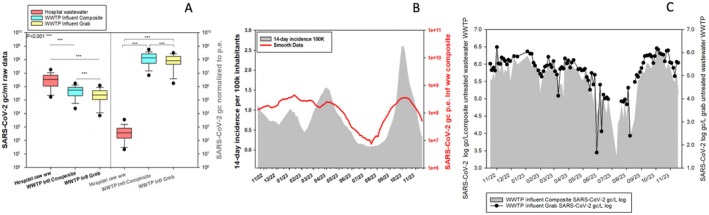
(A) SARS‐CoV‐2 concentrations expressed by genomic copies per mL in untreated WW samples (Hospital ww—red bars; WWTP influent composite—blue bars; WWTP influent grab—yellow bars) and the concentrations normalised to the population equivalent; (B) 14‐day incidence per 100,000 inhabitants in Bucharest (grey area) and SARS‐CoV‐2 concentration normalised to population equivalent in the inflow composite samples from the WWTP (red smooth line); (C) SARS‐CoV‐2 concentration expressed in log genomic copies per litre in untreated WW samples collected from WWTP: Composite (grey area) and grab (line and scatter).

Figure 1A displays the raw monitoring data (SARS‐CoV‐2 genomic copies/mL untreated WW) and the normalised results to the population equivalent (p.e.). The raw data exhibit a general trend, with a higher concentration of SARS‐CoV‐2 gc/mL in the hospital WW, followed closely by the composite sample from the WWTP and one order of magnitude difference for the grab sample from the WWTP. After data normalisation to population equivalent, the concentration of SARS‐CoV‐2 gc/mL drops drastically, as data is normalised to the patients from the hospital. Notably, the SARS‐CoV‐2 results normalised to p.e. in the WWTP do not present a significant difference between the two samples: composite and grab.

During the monitoring campaign, we retrieved epidemiological data from a public website (official data provided by the Romanian public health authorities), represented by 14‐day incidence per 100,000 inhabitants. Compared with SARS‐CoV‐2 genomic copies normalised to population equivalent in the untreated composite WW samples from the WWTP, they both follow a similar trend (Figure 1B). Noteworthy, for the highest peak (October‐November 2023) registered during the monitoring campaign, the increased trend was firstly observed in the SARS‐CoV‐2 concentration in WW and after approximately 2 weeks in the 14‐day incidence per 100 k inhabitants. This pattern is explained firstly by asymptomatic or mildly symptomatic patients who do not report or test themselves as having COVID‐19, and secondly by population movement and migration (e.g., students, residents from Bucharest travelling to neighbouring villages, tourists). During the monitoring campaign, the SARS‐CoV‐2 concentration present in grab and composite untreated WW samples collected from the WWTP exhibits a similar pattern, suggesting that both approaches can be used in WW surveillance (Figure 1C).

### 3.2 Phylogenetic Analysis of SARS‐CoV‐2 Isolates

Our results are correlated with the dynamics of SARS‐CoV‐2 variants in 2022 and 2023, followed by global patterns, with several Omicron sublineages emerging [33, 34]. We detected the BA.5 variant until the middle of January 2022. The BQ1 variant appeared at the beginning of December 2022 and was observed in January 2023 as well. The XBB sublineages, including XBB.1 and XBB.1.5, started to gain attention in late 2022, and we detected XBB.1.5 in February, March and April 2023, coinciding with global trends, where this subvariant started to become dominant in multiple regions. All genome sequences generated in this study are available in the GISAID EpiCoV database (https://www.gisaid.org/) [27] under the accession numbers EPI_ISL_19485272—EPI_ISL_19497459. Registered users can access the data under GISAID's terms of use.

**FIGURE 2:**
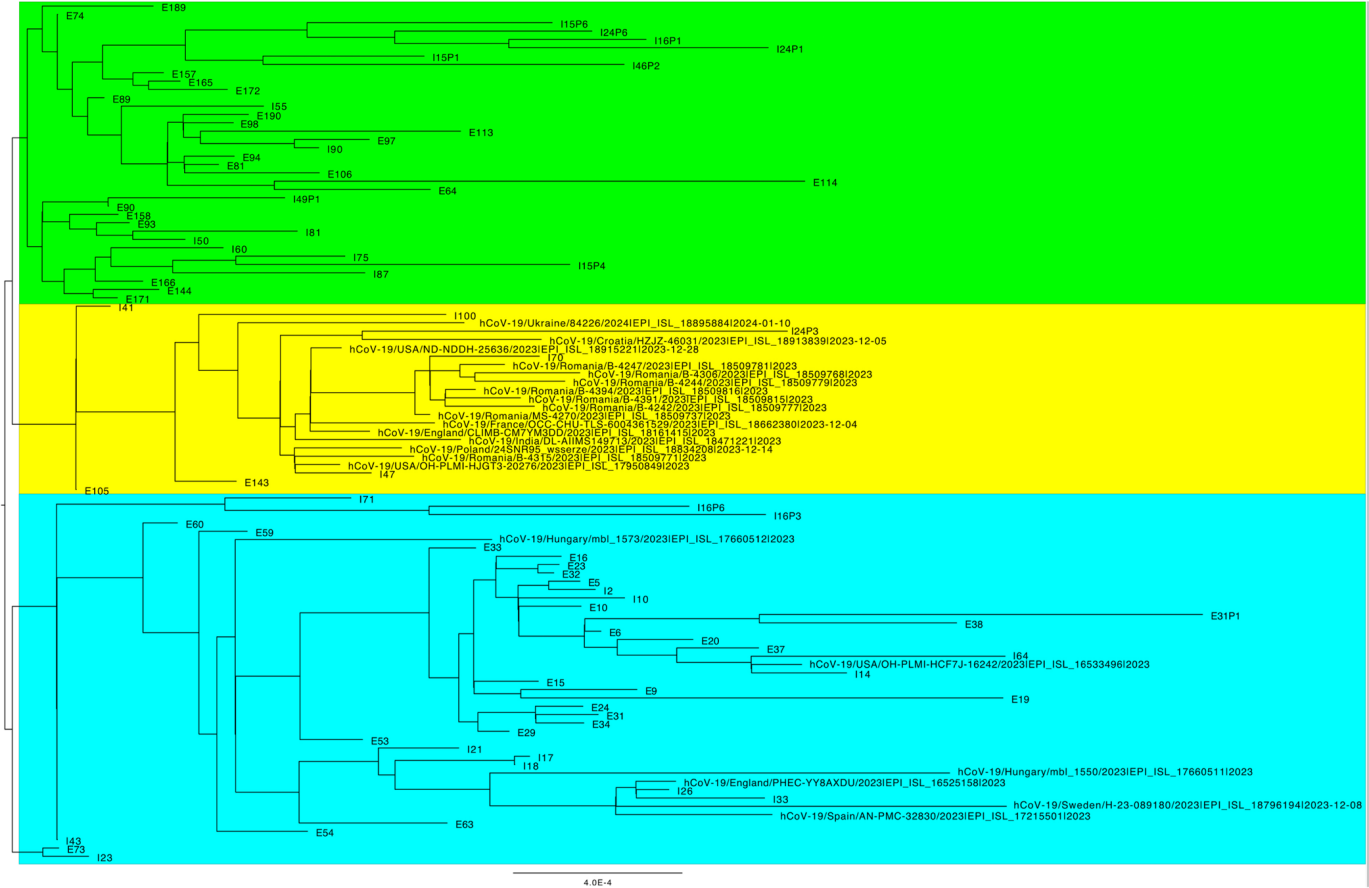
The phylogenetic analysis of SARS‐CoV‐2 isolates from Bucharest WW during the monitoring campaign (I—Hospital grab WW sample; E odd—urban composite WW sample; E even—urban grab WW sample; the number represents the sample ID).

The phylogenetic analysis of SARS‐CoV‐2 isolates revealed the formation of two major clusters, with the predominant cluster further splitting into two subgroups. Notably, one subgroup (highlighted in yellow in the phylogenetic tree image) included sequencing isolates from this study clustered together with all Romanian GISAID sequences, as well as sequences from Croatia, France, Poland, Ukraine, England, the US and India. These sequences belonged to the XBB.2 lineage (XBB.2.3, GE.1, XBB.2.4) from the Hospital grab set of isolates, and XBB.1 (XBB.1, XBB.1.5) from the WWTP composite set. This subgroup exhibited a close relation to the other subgroup (highlighted in green), primarily consisting of sequences from the XBB.1 lineage (XBB.1, XBB.1.16, XBB.1.42, XBB.1.5, XBB.1.9, FL.15, HF.1), with no other GISAID BLASTed sequences assigned to this group (Figure 2). Conversely, the other major cluster comprised SARS‐CoV‐2 GISAID sequences from Hungary, Spain, Sweden, England and the US, along with Romanian sequences obtained in this study. This group was predominantly composed of B.1.1.529 SARS‐CoV‐2 isolates, with no observed correlations to cycle threshold values or sample sources.

### 3.3 Detection of Other Respiratory Viruses in WW

**FIGURE 3:**
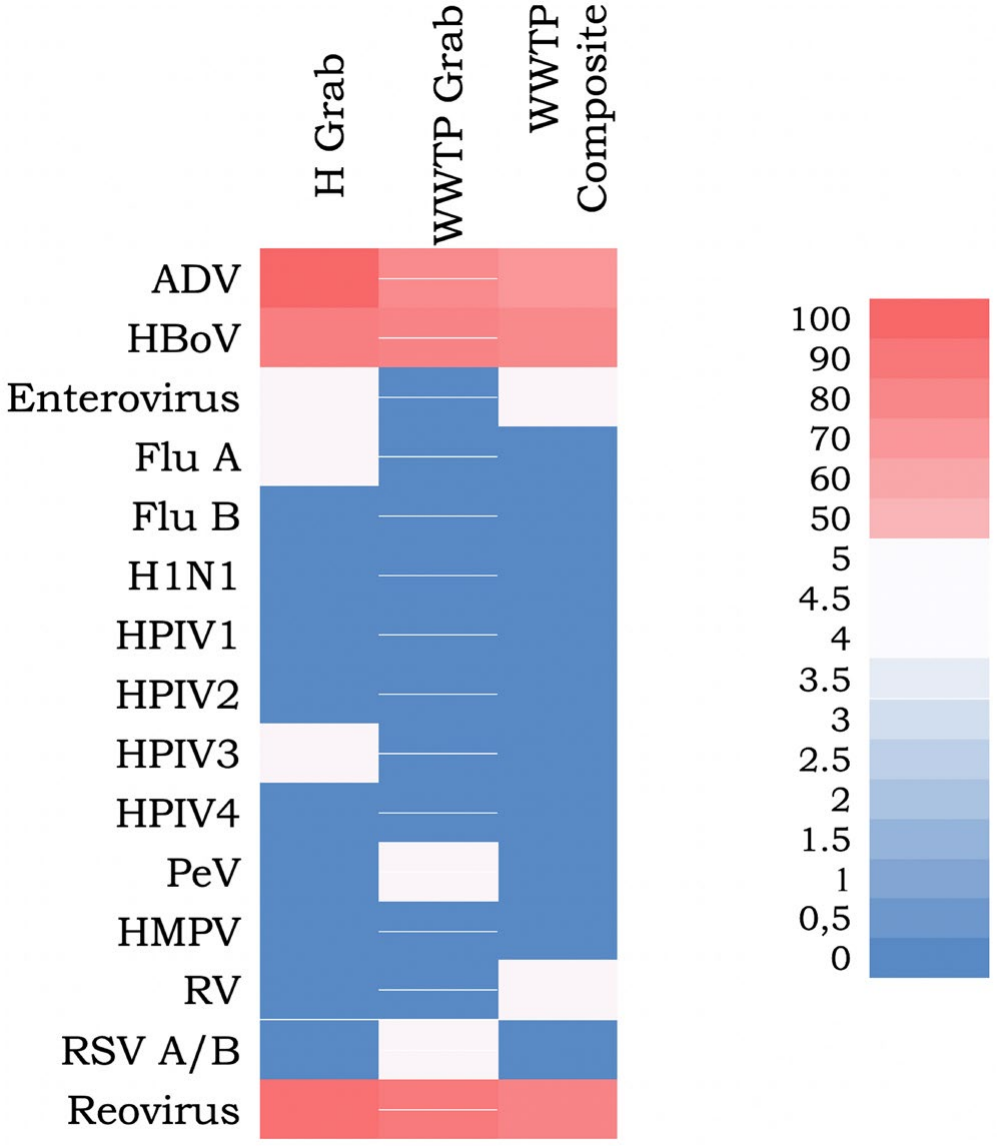
Heat map describing the percentage of WW samples (H grab sample; WWTP grab and composite samples) that were positive/negative for the presence of other non‐SARS viruses. The colour scale shows the meaning of the colours used: Red indicates high values and blue indicates low values. Respiratory Pathogens Panel identifies Pandemic H1N1, RSV A/B, Parainfluenza 3 (HPIV3), Human Metapneumovirus (HMPV), Enterovirus, Adenovirus (ADV), Bocavirus (HBoV), Rhinovirus (RV), Parainfluenza 1 (HPIV1), Parainfluenza 2 (HPIV2), Parainfluenza 4 (HPIV4), Influenza A (Flu A), Parechovirus (PeV), Influenza B (Flu B). Reovirus evaluation was done using nested PCR.

Adenovirus was present in almost all WW samples collected from the hospital. On the other hand, in hospital WW samples, Bocavirus was detected in 72% of the samples, except for the September–November 2023 period. Other viruses were observed randomly in hospital WW: enterovirus in November 2022, Influenza virus in October 2022 and Parainfluenza virus in June 2023. Regarding the WW samples collected from the WWTP, 76% of the grab samples display the presence of adenoviral DNA, with two periods of negativity: March–May 2023 and September–October 2023. Bocavirus was also not present during the period of March–May 2023, but it was detected in September–October 2023. Other viruses detected in the current sample from the treatment plant were RSV (November 2022) and Parechovirus (January 2023). It should be noted that Pneumovirus, Rhinovirus and Enterovirus were detected in cell cultures inoculated with untreated grab WW samples collected from the WWTP. This means that the virus concentration in untreated WW is very low, and the viruses can be observed either after amplification in culture or through much more sensitive methods such as dPCR (Figure 3). Moreover, in the composite sample collected from the WWTP, ADV and Bocavirus were detected in a high percentage (64% and 72%, respectively). The profile of ADV presence in the composite sample resembled that of the current sample: ADV was not detected in March–May 2023 and September–October 2023, and Bocavirus was not detected in March–May 2023. Other viruses were detected only after amplification in cell cultures (Enterovirus and Rhinovirus). Reovirus was detected in the majority of untreated WW samples, as follows: in 87.5% of grab samples from the hospital, 81.25% of grab samples and 75% of composite samples from the WWTP.

### 3.4 Cell Culture WW Viruses Isolation

**FIGURE 4:**
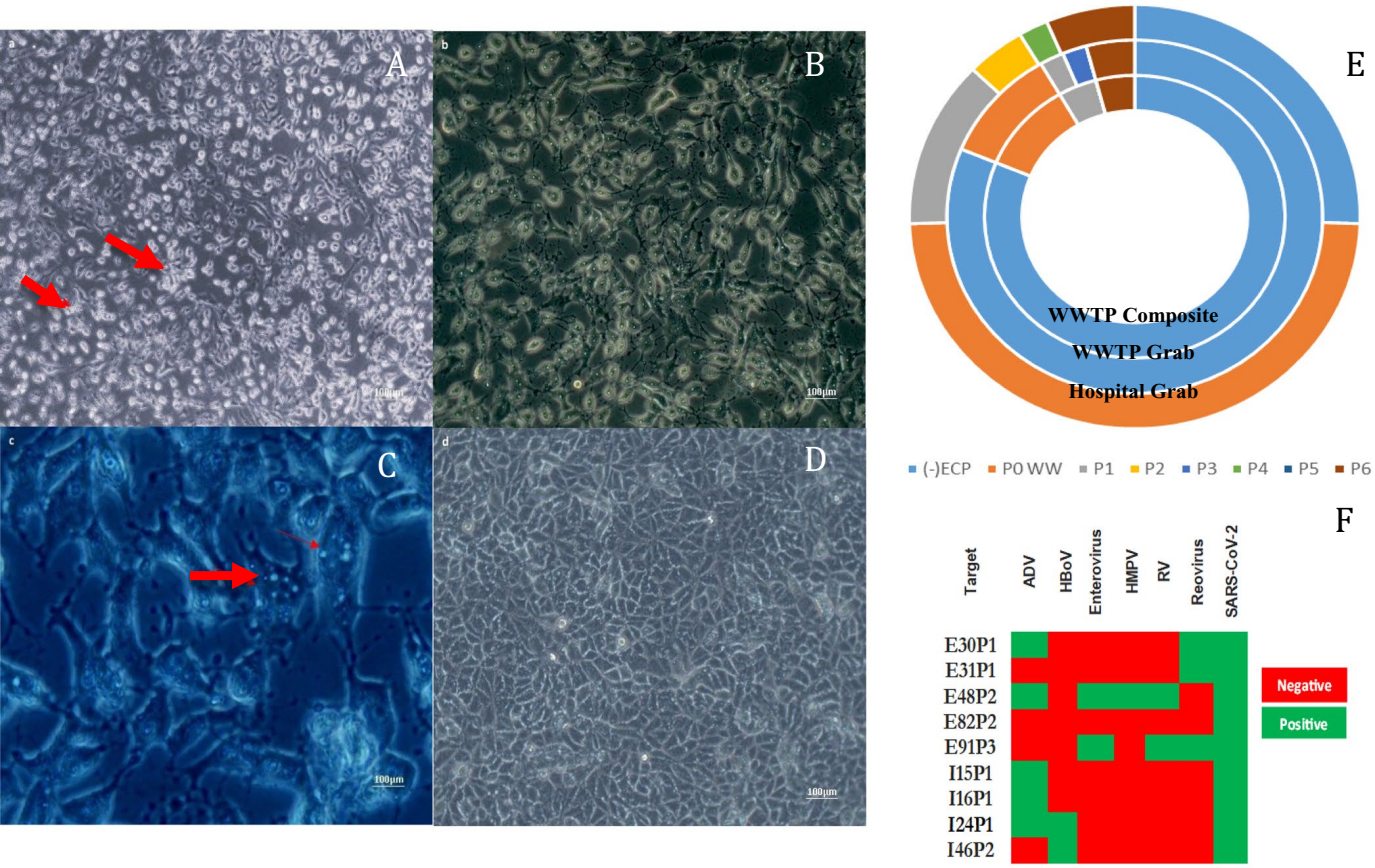
Characterisation of viruses from untreated WW isolated in cell culture: The morphology of cells inoculated with WW viruses. Round pyknotic cells with few aggregates (arrows) due to DNA stickness: Ob. 10× (A); Ob. 20× (B); visible cytoplasmic vacuoles appear as empty vesicles inside cells, as marked by arrows: Ob. 63× (C); VeroE6 uninoculated cells Ob. 20× (D); The scale bar is 100 μm; (E) passage history (−) ECP—without ECP; P0 WW (ww = wastewater)—initial wastewater sample; P1—passage1; P2—passage 2; P3—passage 3; P4—passage 4; P5—passage 5; P6—passage 6. (F) identification of viruses isolated in cell culture (I—Hospital grab WW sample; E odd—urban composite WW sample; E even—urban grab WW sample; the number represents the sample ID).

The VeroE6 monolayers inoculated with filtered viral suspension obtained from WW were observed daily for over 5 days. Changes or lesions observed in the morphology of host cells infected with a viral suspension quantified as a CPE were compared to those in an uninoculated culture. Thus, in VeroE6 cell cultures inoculated with viral suspensions from wastewater, cell rounding was frequently observed, infected cells tending to lose their normal morphology (elongated, polygonal) and becoming round and swollen, and in advanced stages of infection, detaching from the cell substrate, which indicates cell destruction and death, probably associated with the release of viral particles. The viruses caused a loss of intercellular contact, leading to a pronounced separation of cells that would normally be adjacent. Additionally, we observed the aggregation of round pyknotic cells, characterised by the DNA exhibiting stickiness (Figure 4A). In some cases, the appearance of CPE with pyknotic cells occurred within less than 48 h. The presence of visible cytoplasmic vacuoles, appearing as empty vesicles within cells and associated with viral replication and cell autolysis, was frequently observed as SARS‐CoV‐2 CPE (Figure 4B,C). Syncytia, multinucleated giant cells formed via spike protein‐mediated fusion, were not observed in viral isolates from WW.

Untreated WW samples, collected from the WWTP (48 composite and 48 grab samples) and 47 grab samples from the hospital, were used to isolate the virus on cell cultures. A high percentage, 81.25%, of all untreated WW samples collected from the WWTP did not induce a CPE on cell cultures. The remaining 19.75% of the samples were positive, 10.42% showing a CPE only in the initial sample, and 8.33% passing several passages in the cell culture (between 1 and 6 passages). In the case of WW samples collected from the hospital, the percentage of those that did not show a CPE was significantly lower, at 25.53%. Conversely, 48.94% of the samples displayed a CPE only in the initial sample. It should be noted that the infectivity of SARS‐CoV‐2 decreased with increasing passage number, and few viral isolates passed beyond passage 5 from both locations. Figure 4E presents the cumulative data of the isolates from untreated WW samples.

### 3.5 TEM Analysis

**FIGURE 5:**
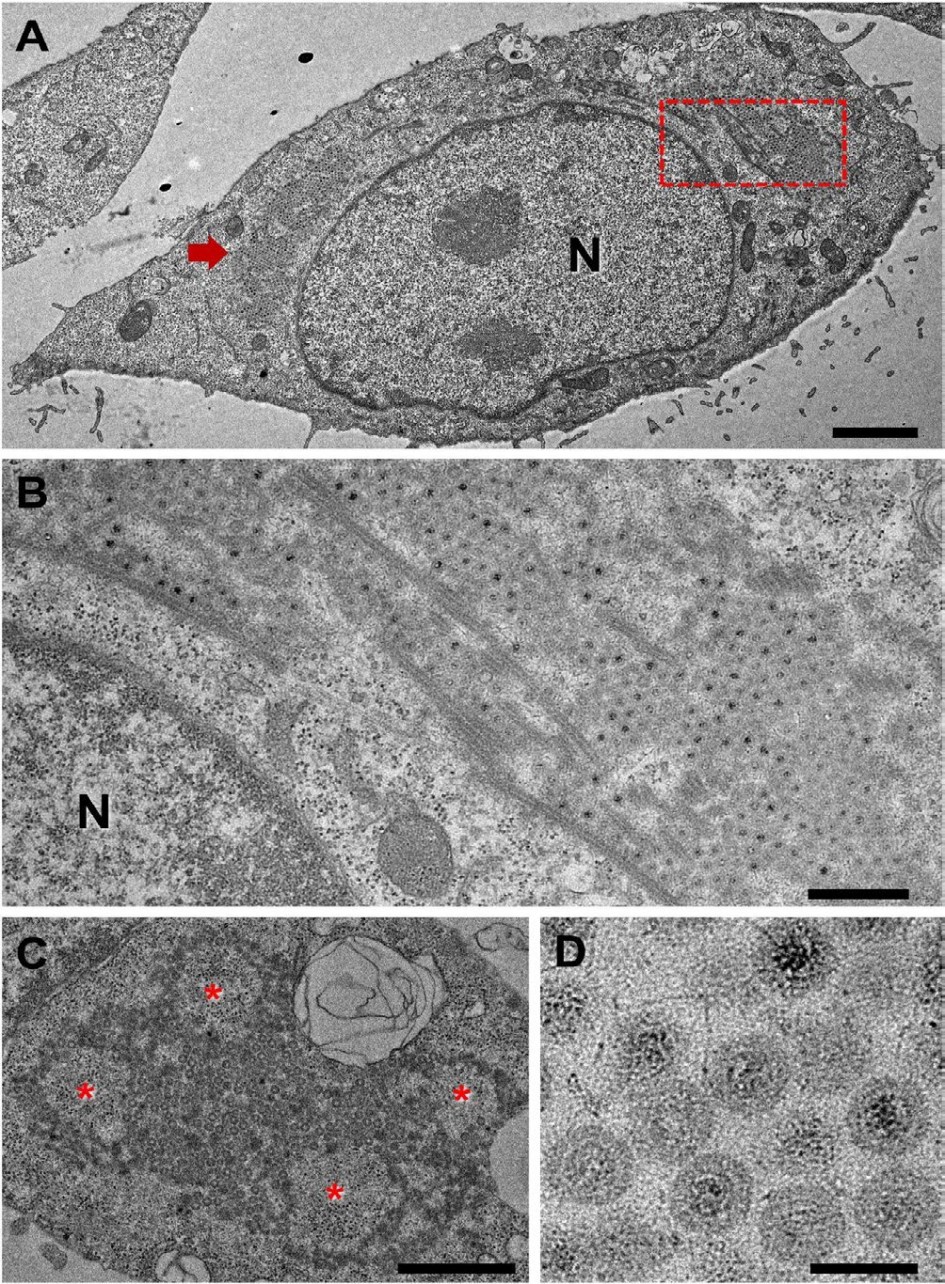
Reovirus‐like particles. (A) Cells presented frequent clusters of spherical virions occupying large areas of the cytoplasm (red arrow and boxed area). Scale bar is 2 μm. (B) Higher magnification of boxed area in (A), showing these clusters associated with microtubule networks. Scale bar is 500 nm. (C) Virion clusters are also expanded around islands of cytoplasm rich in ribosomes (red asterisks). Scale bar is 1 μm. (D) Individual virions had relatively constant diameters (80–85 nm) and some had a visible electron‐dense core, features highly suggestive of reoviruses. Scale bar is 100 nm. N—nucleus.

**FIGURE 6:**
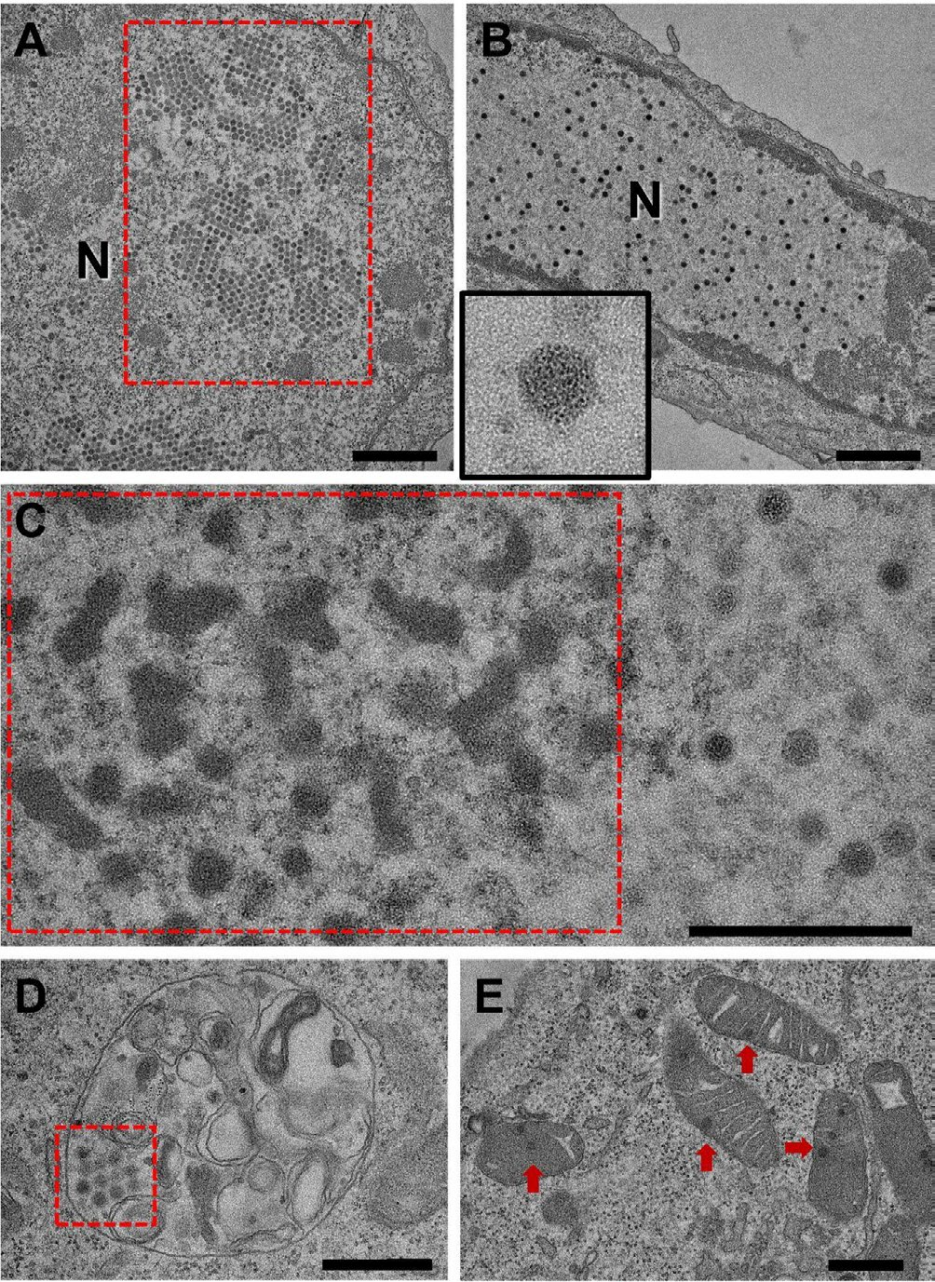
Adenovirus‐like particles. (A) Some infected cells presented extensive nuclear paracrystalline arrays (red box), consisting of virions at different stages of maturation. (B) In other cells, these virions appeared randomly distributed in regions of euchromatin. These virions were between 65 and 90 nm, lacked envelopes and presented icosahedral symmetry, features highly suggestive of adenoviruses (inset, box size 200 × 200 nm). (C) Nuclei of infected cells also presented polymorphic viral inclusions associated with viral assembly (red box). Virions were frequently found outside the nucleus, predominantly in endolysosomes (D, red box) and mitochondria (E, red arrows). Scale bar for (A) and (B) is 1 μm, all other scale bars indicate 500 nm. N—nucleus.

We performed TEM to observe ultrastructural changes associated with VeroE6 cell infection following WW treatment. Overall, non‐apoptotic treated cells frequently exhibited distended rough endoplasmic reticulum cisternae and displayed more cytoplasmic single‐membrane vesicles than untreated cells. The most striking feature in approximately 20% of analysed cells were cytoplasmic inclusions (viroplasms) with numerous reovirus‐like particles and associated microtubules (Figure 5A,B). Viroplasms surrounded regions of the cytoplasm, rich in ribosomes, likely driving viral replication (Figure 5C). The spherical virions were 80–85 nm in diameter and presented as either empty shells or with an electron‐dense core (Figure 5D). A separatevirus population, consisting of 65–90 nm virions with symmetrical capsids, localised within euchromatin‐rich regions of the nucleus, forming paracrystalline arrays typical of adenovirus infection (Figure 6A–C). In cells presenting these nuclear arrays, virions were also found in endolysosomes (Figure 6D), free in the cytoplasm (data not shown), and, interestingly, in most mitochondria, which showed disruption of the cristae structure and vacuolization (Figure 6E).

**FIGURE 7:**
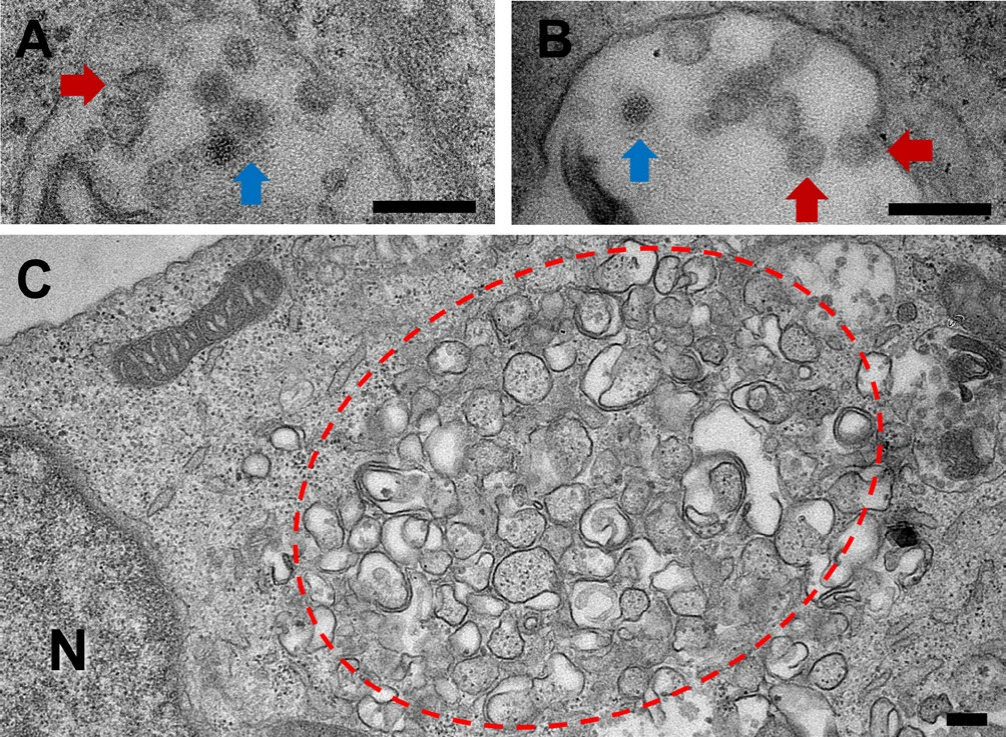
SARS‐like particles. Definitive identification of SARS‐CoV‐2 was not possible, despite the presence of some suggestive features such aslarger diameters, a lipid bilayer, surface spike‐like protrusions or nucleocapsid cross‐sections (red arrows in A,B). These SARS‐like particles were differentiated from other types of potential virions, which were smaller (< 90 nm), lacked an envelope and had evident internal symmetry (blue arrows in A,B). Double membrane vesicles were observed very rarely and appeared devoid of viral particles (C, circled area). N—nucleus. All scale bars indicate 200 nm.

Finally, some cells from samples with PCR‐confirmed SARS‐CoV‐2 infection exhibited cytoplasmic vacuoles containing SARS‐like particles; however, unequivocal visual identification of SARS‐CoV‐2 proved challenging. This was due to the rarity of potential virions and poor preservation of characteristic features, including surface spikes and internal densities suggestive of ribonucleoproteins (Figure 7A–D). Extracellular viral particles were absent at the analysed time points. Moreover, we could identify only two cells with double membrane vesicles, indicating early‐stage or low‐grade SARS‐CoV‐2 infection.

### 3.6 Identification of SARS‐CoV‐2 Variants and Non‐SARS Viruses From Viral Isolates Obtained in Cell Cultures From Untreated WW Samples

Sequencing data revealed SARS‐CoV‐2 variants isolated in the cell culture: the isolates from December 2022 were BQ1, those from January 2023 were included in the BA group, and those from March 2023 in the XBB 1.1.6 group; so that later in April, we detected the XBB1.5 variant. Our findings are consistent with global patterns of SARS‐CoV‐2 variant evolution, highlighting how WW surveillance can be a useful tool for tracking viral mutations in a community [33]. Several viruses, including Adenovirus, Reovirus, Enterovirus, Pneumovirus, Bocavirus and SARS‐CoV‐2 (Figure 4F) were identified in cell culture. However, viral diversity is probably underestimated in our isolates, which metagenomics could reveal more fully.

## 4 Discussion

We quantified SARS‐CoV‐2 RNA in WW using RT‐qPCR at a hospital and WWTP sites. The highest concentration was in the hospital grab sample (5.63E+06 gc/L), followed by the WWTP composite (6.23E+05 gc/L) and grab sample (3.71E+05 gc/L). The minimum concentration of SARS‐CoV‐2 RNA was detected in the WWTP grab sample, highlighting the importance of the dilution factor, the timing of sample collection and population mobility. Although raw data showed higher concentrations in hospital WW, after data normalisation to population equivalent, the concentration decreased drastically as the data was normalised to hospital patients, demonstrating once more the utility of WBE. Notably, the highest viral peak in WW (October–November 2023) preceded the reported case incidence peak by approximately 2 weeks, with an incidence of 14 days per 100,000 inhabitants, which is in line with other studies worldwide [11, 12]. The monitoring of SARS‐CoV‐2 in WW, correlated with epidemiological data, makes a significant contribution to the public health system by anticipating increases in the actual number of cases, encompassing both pre‐ or asymptomatic individuals.

Similar to our data, at the beginning of 2023, newer variants, such as BQ.1 and XBB.1, were observed. XBB.1 subvariants showed growth advantages and immune escape capabilities, similar to other co‐circulating lineages at that time [35, 36]. For example, XBB.1.16 and related variants have presented an increased incidence worldwide, including in Europe, due to their rapid growth in cases, although they have not significantly increased severity or hospitalisations compared to previous Omicron waves [37, 38, 39]. The observations align with Romania's case, where those specific subvariants did not cause a drastic rise in severe cases, indicating a continuation of Omicron's trend of higher transmissibility but lower disease severity compared to earlier variants, such as the Delta variant [33, 40, 41]. XBB.1 displayed a gradual rise in case numbers, with peaks in different regions during the first quarter of 2023, coinciding with a wave of increased COVID‐19 infections [42]. The transition from XBB.1 to newer variants generally occurred by mid‐2023, with XBB.1.5 and other related lineages, such as XBB.1.9.1 and XBB.1.16, becoming more prevalent [43]. The evolution of SARS‐CoV‐2 variants and their dissemination reflect the ongoing changes in virus transmissibility, immune escape and population immunity levels from vaccination and previous infections [33, 34].

WBE detects viral genetic material in wastewater, offering an indirect measure of population‐level prevalence and enabling early warning of viral spread before clinical symptoms appear. In contrast, virus isolation in cell culture requires the presence of a viable virus in the sample [44], which can infect susceptible cells, replicate and be successfully isolated, indicating that an active infectious virus exists in the sample, not just non‐viable RNA fragments [45, 46, 47]. Therefore, virus isolation in culture complements the epidemiological data from WW, demonstrating that the virus detected is viable and can contribute to disease transmission in the community [3, 48]. The present data show that a few SARS‐CoV‐2 isolates from WW samples can be maintained in culture for several passages, but we cannot establish the degree of infectivity.

In summary, a CPE of SARS‐CoV‐2 was observed in cell culture and subsequent passages, confirmed by RT‐PCR. However, a key role in demonstrating the infectivity of SARS‐CoV‐2 isolated from WW is played by the competitiveness rate, given that untreated WW contains a high concentration of viruses from different classes, including enveloped and non‐enveloped viruses, which exhibit distinct properties (such as detection, survival, resistance and adaptation). On the other hand, WW samples contain many inhibitors (potentially toxic) for eukaryotic cell cultures and a high diversity of viral particles. Due to the increased competitiveness of viruses and WW inhibitors content, a direct relationship could not be established between viable SARS‐CoV‐2 in WW and public health risk (e.g., persons who operate the WWTPs). Although TEM was helpful in identifying ultrastructural features characteristic of co‐infection with at least two other virus species, morphological changes associated with SARS‐CoV‐2 replication were discrete and limited to an increase of cytoplasmic single‐membrane vesicles [49] and very rare double membrane vesicles. Additionally, the low number of putative intracellular virions and the absence of extracellular virions at the selected time points suggest that SARS‐CoV‐2 was outcompeted for the cell's replication machinery by other WW pathogens.

The in vitro phenomenon of multiple viruses infecting the same cell culture simultaneously may reproduce the in vivo viral co‐infection in human hosts that falls under three major virus‐virus interactions: viral interference (one virus suppresses another), viral synergy (co‐infection stimulates replication of both viruses) and viral noninterference (viruses coexist without affecting each other's replication) [50]. The variability of SARS‐CoV‐2 viral isolates refers to the genetic (increased rate of mutations leading to the emergence of new viral variants or strains) and behavioural differences between different strains of the virus, which may influence the virus's ability to be cultured in cell cultures, with some strains requiring specific culture conditions and having a reduced ability to infect or multiply in certain cell types. The exact mechanisms of simultaneous SARS‐CoV‐2 infection with other viruses are complex and will be analysed in depth in future studies. Further studies that incorporate the use of multiple cell lines, both at the initiation and during the subculturing of viruses, can enhance detection probabilities based on the different affinities of viruses for specific cell lines. Implementing these strategies, including WBE and cell culture isolation, allows us to conduct a more precise assessment of public health risks.

From a public health standpoint, the presence of viable virus in wastewater, even at low levels, warrants attention, particularly in settings with inadequate sanitation or direct human exposure to contaminated water. While wastewater is unlikely to be a dominant transmission route, its role as a reservoir for viral genetic material and a matrix for viral interaction deserves further investigation. Continuous monitoring and improved understanding of wastewater virology could help assess environmental risks and inform mitigation strategies during current and future outbreaks.

## Author Contributions

**Elena Radu:** conceptualization (equal), data curation (equal), formal analysis (equal), investigation (equal), methodology (equal), validation (equal), writing – original draft (equal), writing – review and editing (equal). **Tudor Emanuel Fertig:** investigation (equal), visualization (equal), writing – original draft (equal). **Laura Denisa Dragu:** investigation (equal), visualization (equal). **Ioana Mădălina Pitică:** investigation (equal), visualization (equal). **Marius Surleac:** formal analysis (equal), visualization (equal), writing – original draft (equal). **Ana Iulia Neagu:** investigation (equal), visualization (equal). **Lavinia Pană:** investigation (equal), visualization (equal). **Alexandra Păiş:** investigation (equal), visualization (equal). **Lilia Matei:** investigation (equal), visualization (equal). **Ionut‐Lucian Antone‐Iordache:** investigation (equal), visualization (equal). **Daciana Silvia Marta:** investigation (equal), visualization (equal). **Victor‐Eduard Peteu:** investigation (equal), visualization (equal). **Mihai Niţă‐Lazăr:** investigation (equal), visualization (equal). **Cătălina Stoica:** investigation (equal), visualization (equal). **Cornel Popescu:** investigation (equal), visualization (equal). **Camelia Mădălina Sultana:** investigation (equal), visualization (equal). **Anca Botezatu:** investigation (equal), visualization (equal). **Iulia Virginia Iancu:** investigation (equal), visualization (equal). **Mihaela Chivu‐Economescu:** investigation (equal), visualization (equal). **Leontina Banică:** investigation (equal), visualization (equal). **Ana Sorinica Petre:** investigation (equal), visualization (equal). **Simona Paraschiv:** investigation (equal), visualization (equal). **Mihaela Gherghiceanu:** investigation (equal), validation (equal), visualization (equal). **Simona Maria Ruta:** validation (equal), visualization (equal), writing – review and editing (equal). **Norbert Kreuzinger:** methodology (equal), visualization (equal). **Carmen Cristina Diaconu:** funding acquisition (equal), project administration (supporting), resources (lead), supervision (equal), validation (equal), visualization (equal), writing – review and editing (equal). **Coralia Bleotu:** conceptualization (equal), data curation (equal), formal analysis (equal), investigation (equal), methodology (equal), supervision (equal), validation (equal), visualization (equal), writing – original draft (equal), writing – review and editing (equal).

## Conflicts of Interest

The authors declare no conflicts of interest.

## Data Availability

All genome sequences generated in this study are available in the GISAID EpiCoV database (https://www.gisaid.org/) [27] under the accession numbers EPI_ISL_19485272‐EPI_ISL_19497459. The data can be accessed by registered users in accordance with GISAID's terms of use.
